# Electricity and cryptocurrency mining: An empirical contribution

**DOI:** 10.1016/j.heliyon.2024.e33483

**Published:** 2024-06-24

**Authors:** David Iheke Okorie, Joel Miworse Gnatchiglo, Presley K. Wesseh

**Affiliations:** a Hangzhou City University (HZCU), Hangzhou, China; b University of Waikato, Management School, School of Accounting, Finance and Economics, Hamilton, New Zealand; c SD Consulting Agency (SCA), Lagos, Nigeria; d Center for African Department Strategy (CFADS), Monrovia, Liberia; e School of Management, China Institute for Studies in Energy Policy, Collaborative Innovation Center for Energy Economics and Energy Policy, Xiamen University, Fujian, 361005, China

**Keywords:** Electricity consumption, Cryptocurrency, Return, Volatility spillover

## Abstract

Active cryptocurrency mining and trading comes with heavy electricity demand and increased emissions. Thus, cryptocurrency mining is prohibited in most economies. Consequently, miners relocate to regions or economies without these prohibitions and/or with relatively lower electricity rates. As such, presenting a nexus between the cryptocurrency and electricity markets, even at the global level. This article investigates the different forms of relationships existing between these markets. The conditional asymmetric volatility model with the Wald, nonparametric and parametric Granger causality tests are employed. The results confirm the existence of both unidirectional and bidirectional lead-lag return relationships between the cryptocurrency and electricity markets. Cryptocurrency returns drive electricity demand. This finding is homogeneous both on a global and strata (homogeneous groupings) basis. Also, the electricity market spills over significant volatilities to the cryptocurrency markets without feedback, nonetheless. Result-based policies are recommended towards green finance, decarbonization, and emission mitigations through the demand for electricity by the cryptocurrency markets. They include the use of clean and renewable electricity sources and technologies for cryptocurrency market activities.

## Highlights

• This paper investigates the Electricity and Cryptocurrency markets.
• Explores parametric & nonparametric lead-lag and transmission effects.
• Cryptocurrency returns drive the demand for electricity market.
• The electricity market spills over volatilities to the cryptocurrency markets.

## 1 Introduction

Global technological progress, inventions, innovations, and development are substantially linked to energy consumption. These encourage the rapidly growing demand for electricity in various economies [1]. Consequently, more carbons are emitted. These developments include that of the cryptocurrency markets. Specifically, the active mining and trading activities of these cryptocurrencies require huge electricity use. Studies have established substantial information spillovers between electricity and cryptocurrency markets [2]. Therefore, this article goes a step further to investigate several forms of relationships existing between the electricity market and the development of the cryptocurrency market. Bitcoin and Ethereum are taken as representatives for the cryptocurrency markets given their popularity, price developments, investor base (private and institutions), market capitalization, etc. On the other hand, the electricity markets are sampled on a global scale from eight-nine countries. Thus, this paper examines the different forms of connectedness between the global electricity market (89 countries) and the cryptocurrency markets (Bitcoin and Ethereum).

Zhang et al. [3] noted that the cryptocurrency blockchain serves extra advantages to its users and aggregators (cost-saving benefits). Besides, the blockchain system facilitates the machine-to-machine market of electricity [4], blockchain can improve the geo-energy industry's efficiency level [5], blockchain can improve local energy trading such as Brooklyn Microgrid [6], blockchain harnesses micro-grid operations [7], blockchain enables carbon emission trading and applications [8], etc. There are very few related papers that study the relationship between electricity and cryptocurrency markets [9,10]. This article makes novel contributions to electricity demand and cryptocurrency mining on a global and strata scale. Secondly, this study investigates the volatility connectedness between the electric markets and the cryptocurrency markets. That is, this paper examines the connectedness (linear & non-linear lead-lag relationships and volatility transmission effects) between the global and strata electricity market (89 countries) and the cryptocurrency markets (Bitcoin and Ethereum). The results confirm that there exists a bidirectional Granger causality between cryptocurrency (Ethereum and Bitcoin) prices and the Hash rates. There also exists a unidirectional causality from the return of Bitcoin to electricity demand, irrespective of the electricity cost. Moreso, our results show a significant volatility spillover from the electricity market (demand side) to the Bitcoin market. These findings support those of existing studies like Liu et al. [11], Wang et al. [12], Zhang et al. [13], etc. The trading and mining activities of cryptocurrencies and their interactions with an economy [2] and other markets [14] cannot be overemphasized. Besides, they are prone to external shocks like other markets [15,16]. Despite these, the return & volatility of cryptocurrency markets, like the stock markets, are predictable given the right factors and methods [[17], [18], [19]].

### 1.1 Why electricity and crypto markets?

Globally, the past five decades have recorded substantial increases in the level of electricity consumption.^1^ For instance, the worldwide electricity consumption level (net) in 1980 was around 7323 billion kilowatt-hours. By the next decade, in 1990, approximately a 42 % increase was recorded in the level of electricity consumption (per hour) globally. In 2000, the estimated level increased to 13,277 billion kilowatt-hours. Recall that before the end of the next decade, i.e. 2010, cryptocurrencies kicked in while their recognition and development kept increasing over the years. About 18,640 billion kilowatt-hours were recorded in 2010 as the electricity consumption level and by 2017 it had tremendously increased by 20 % approximately. In 2018, China, the United States, India, Japan, and Russia were ranked as the top 5 economies based on electricity consumption (in the 2019 Global Energy Statistical Yearbook). These economies are also the leading economies in mining cryptocurrencies. The World Energy Outlook stipulates that the global reliance on fossil fuels would increase substantially in 2020 and even in the future.

The linkages between the energy (electricity) market and the cryptocurrency markets hinge on the electricity demand for the mining and active trading of cryptocurrencies. Cryptocurrency mining and trading are energy-intensive due to the technologies needed for trading and mining cryptocurrencies. In simple terms, participating or being an investor in the crypto markets and exchanges requires that the investor (often at all times) study and monitor the price fluctuations of these cryptocurrencies to decide when it is best to take short and long positions. For cryptocurrency miners, the mining machines and algorithms more or less run at all times. To do these, the technologies required a range from as small as mobile cell phone apps to as big as heavy mining machines. The use of mobile cell phone apps for cryptocurrency market trading as well as the demand for heavy cryptocurrency mining machines requires the use of electricity. This increased electricity consumption in an economy is directly proportional to the concentration of crypto market investors or traders and miners and it is expected to be inversely related to the electricity rate or price. Therefore, the electricity demand for cryptocurrency trading and mining is no longer a problem of one or a few economies but a global issue given that miners could move from regions of higher relative electricity rates to regions of relatively lower electricity rates. Similarly, the exchanges operate globally too.

**Fig. 1:**
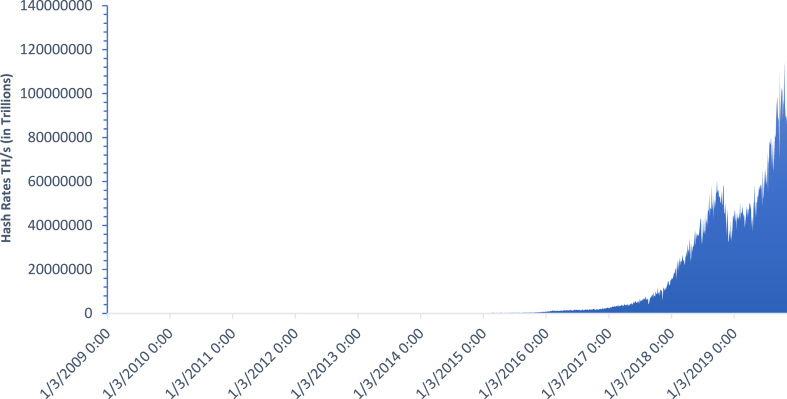
Bitcoin Hash Rate, measured in Tera Hashes per Second.

**Fig. 2:**
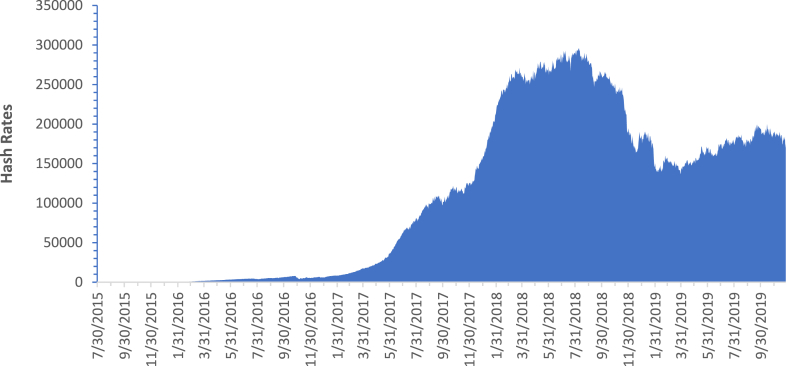
Ethereum Hash Rate, measured in Hashes per Second.

For instance, heavily concentrated cryptocurrency market investors like the USA, China, Japan, etc have relatively higher electricity utility charges and hence, miners and exchanges go global and establish mining facilities in economies with relatively fewer electricity charges. Therefore, the impact of electricity demand or consumption on the cryptocurrency markets is not limited to heavily cryptocurrency investor countries but stretches to the (relatively) cheaper electricity utility charge countries (see Figure 1 in Okorie [2]). The trillions of hashes per second (over the years) shown in Figures 1 and 2 correspond with the increased cryptocurrency mining rate. The Hash rates capture the processing power of cryptocurrencies as they are mined. Notwithstanding, it is important to point out that about 60 %–80 % of the revenue realized from cryptocurrency mining goes back to paying the bills for the amount of electricity used. Hence, miners look out for cheaper electricity-rate-regions to establish their mining businesses and maximize profits [20]. In 2018, Elite Fixtures conducted a study in 115, countries using government data on the mining cost of Bitcoin relative to average electricity cost.

The results show significant differences in the cost of mining 1 Bitcoin, based on average electricity rates across 115 countries [21]. Based on the mining cost per country as presented in Reiff [21] confirms that the cost of mining a Bitcoin is as high as $26,170 in South Korea, $17,566 in Niue, $16,773 in Bahrain, $16,209 in the Solomon Islands, Cooks Islands, etc. while it is as cheap as $1190 in Trinidad and Tobago, $1788 in Uzbekistan, $1852 in Ukraine, $1983 in Kuwait and Myanmar, etc. Besides, these demands for electricity include other needed gadgets like the air conditioners needed to cool the mining machines, etc. Therefore, economies in the coastal regions will still not consume more electricity, relative to other economies not in coastal regions given similar electricity rates. Based on this distribution, the countries are grouped into different quantiles from lower electricity cost mining countries to higher electricity cost mining countries, construct an electricity demand or consumption index for each quantile, and examine the relationships between the electricity consumption or demand index for these groups of countries with the Cryptocurrency markets (Bitcoin and Ethereum) concerning the existence of a lead-lag relationship, and return & volatility spillovers effects.

While the mining machines form the fixed costs, the electricity use forms the variable cost [22]. As such, cost minimization entails that cryptocurrency mining activities are taken to economies with cheaper electricity costs. Given that countries with higher concentrations of cryptocurrency active traders or investors might not have the best (cheapest) electricity charges thus, the miners are induced to move to other economies with relatively lower electricity rates to minimize costs. The Miners' revenue is indirectly related to the energy consumption of electricity [23]. Firstly, the lead-lag relationships (both linear and nonlinear) between the cryptocurrency prices & the hash rates, and between cryptocurrency returns & electricity demand are examined. Secondly, the 89 countries are grouped into different homogenous groups or strata based on their electricity rates [21], the electricity consumption indexes for these homogenous groups are constructed, and the return and volatility relationships between the electricity market (demand side) and returns from the markets for cryptocurrency are examined. In a nutshell, given that cryptocurrency active mining, as well as trading, requires high electricity demand which comes with varying costs (based on different economies) for the same good, cryptocurrency. Coupled with the fact it is expected to sell at the same price (law of one price). This paper seeks to examine the volatility spillovers and lead-lag relationships between the cryptocurrency markets (Bitcoin & Ethereum) and the electricity market (demand side). Besides, the lead-lag links between crypto prices and mining (using the hash rates) are equally investigated in this study.

## 2 Summary of relevant studies

Studies like Li et al. [10] and Menatai [24] have shown significant effects of cryptocurrency mining on the electricity market through their consumption of electricity. These studies have adopted both experimental approaches and simulation design approaches. That being said, electricity is essential for improving living standards [25,26]. Csereklyei [27] estimated the residential (industrial) elasticity demand of the European Union (EU) to be between 53 % and 56 % (75 % and 101 %). For selected 132 countries, the long-run industrial (residential) electricity demand elasticity is 88 % (78 %) [28]. Whereas, it's a 72 % residential electricity demand for the Organization for Economic Co-operation and Development (OECD) countries on average [29]. Guang et al. [30] investigated the impact tariffs have on the demand for electricity of the residents in China (Zhejiang Province). Similar studies exist for other economies, such as the West African countries [31], Europe [32], Greece [33], United States [34], Pakistan [35], Italy [36], Spain [37], Portugal [38], Jamaica [39], Australia [40], Japan [41], Sweden [42], etc. The uncertainties and electronic waste generation by the cryptocurrency market have been explored [43,44]. On the other hand, Fan et al. [45] evaluated climate change impacts on electricity demand. MacMackin et al. [46] studied the weather effects on sectoral demand for electricity.

Different cryptocurrency exchange platforms are in different currencies and countries trading the same cryptocurrencies. Given the law of one price, these prices must be equal after taking into account the direct and indirect overhead costs involved in trading on these different exchanges as well as the currency exchange rates. This is theoretically expected to be the case to minimize or mitigate speculation activities in the cryptocurrency markets. Researchers have made tremendous efforts to study the exchanges with the leading information set which determines the cryptocurrency prices. Brandvold et al. [47] show that the Mt.Gox exchange (originated in Japan) and BTC-e exchange (originated in the USA) are the leading exchanges that drive Bitcoin prices. Pagnottoni & Dimpfl [48] noted that the findings of Brandvold et al. [47] became obsolete after the bankruptcy of Mt. Gox on February 28th, 2014. Their study also examined price discovery. But in their case, they included exchanges that trade in US dollars, Chinese Renminbi, and Euro to factor in the exchange rates amongst these currencies into their analysis. They considered Bitfinex (originated in the British Virgin Islands), Bitstamp (originated in Luxembourg), and BTC-e which trades in US dollars; BTC China (originated in China) and OKCoin (originated in China), which trades in Chinese Renminbi; and Kraken exchange (originated in the USA), which trades in Euro. Their results show that the OKCoin exchange leads the price discovery of Bitcoin and is followed by BTC China while the exchange rate plays no role in the price discovery of Bitcoin. Presently, BTC China has liquidated and therefore rendered these findings obsolete. Giudici & Polinesi [49] discovered that Bitstamp drives the price discovery of Bitcoin and has the highest price information set of Bitcoin. They further added that traditional asset prices do not affect Bitcoin prices but have a significant inverse effect on its volatility. Silantyev [50] shows that the BitMex exchange is the leading exchange that drives Bitcoin prices. These show that these exchanges are becoming popular and attracting more investors, both local investors and international investors. Moreso, the origination of these exchanges from an economy indicates that there is more or less, significant cryptocurrency trading and mining in that economy even though the exchanges tend to operate globally.

While the prices of cryptocurrencies have evolved upward over the years, the mining of cryptocurrencies turned out to be a profitable business thus, leading to an increase in the number of people in the mining industry and thus increasing the hash rates. Deng et al. [51] studied the relationship between Bitcoin prices and Hash rates. They found out that the Bitcoin price granger causes the Hash rates and that the current mining price is determined by past cryptocurrency prices. Goodkind et al. [9] built on the estimated energy use of cryptocurrency mining to estimate the economic damage (air pollution, climate impacts, and human mortality) per coin mining of cryptocurrencies in China and the USA. Their results show that a dollar Bitcoin mined is responsible for $0.49 and $0.37 health and climate damages in the USA and China respectively. Another study in Chelan county, Washington DC, with an influx of cryptocurrency mining, highlights the impacts of the heavy cryptocurrency mining on the local energy supply and rates (prices), the socioeconomic benefits uncertainties in the locality, environmental effects and implications, etc [52]. Küfeoğlua and Özkuranb [53] examined the computational electricity power demand of Bitcoin mining during the proof-of-work process and their results show that the electricity demand for Bitcoin mining is as high as 1.3 GWh to 14.8 GWh. The peak period was December 18, 2017 and this electricity demand is between the installed electricity capacity of Finland (∼16 GWh) and Denmark (∼14 GWh) while the total energy consumption of Bitcoin mining per anum stood between 15.47 TWh and 50.24 TWh. Morley et al. [54] consider internet traffic growth and its implications on energy, electricity, and demand. Their results show that the peak of electricity demand tends to occur during the evening periods and varies over time as a result of the provision and design of online services. Similarly, Stoll et al. [55] compared the electricity demand for Bitcoin to that of Jordan and Sri Lanka per annum, which stands (estimated) at 45.8 TWh with a carbon emissions range of 22.0–22.9 MtCO_2_. Vries [56] estimated Bitcoin's electricity consumption to be, at minimum, 2.55 GW (GW) and it is expected to become 7.67 GW going forward which compares to the electricity demand of Ireland (3.1 GW) and Austria (8.2 GW).

Turby [1] noted that Bitcoin mining is an inefficient use of scarce energy resources at a point where every economy wishes to reduce and control energy use and move to clean energy but his paper explores the chances of promoting environmentally sustainable development of applications for Blockchain technology without affecting the sector adversely and mitigate energy consumption by the blockchain technologies. Hayes [22] highlighted that electricity cost is the major cost of cryptocurrency production and that Bitcoin price can be characterized by its cost of production i.e. electricity demand for mining Bitcoin. Bitcoin's electricity demand per annum is estimated to be 45.8 TWh (synonymous with the electricity demand for Jordan and Sri Lanka) while emitting carbon ranging from 22.0 to 22.9 MtCO_2_ [55]. Li et al. [10] developed a cryptocurrency mining experiment for nine different cryptocurrencies using ten different mining algorithms in the Monero mining framework. The experimental result shows that the mining efficiency of cryptocurrencies is mostly determined by the mining algorithm adopted. They went ahead to estimate the electricity demand for cryptocurrency mining and carbon emissions. They claim that the global mining electricity demand of 2018 was 645.62 GWh while only the Monero mining in China is most likely to consume 30.34 GWh and contribute about 19.42 thousand tons of carbon emission from April to December of 2018.

## 3 Data and models

### 3.1 Data

The data employed for analysis are the electricity demand series for 89 countries^2^ with a significant amount of cryptocurrency trading and mining within the sampled periods, the daily price series of Bitcoin (from April 28, 2013 to November 26, 2019) and Ethereum (from August 7, 2015 to November 26, 2019), the daily Hash rates of Bitcoin (from April 28, 2013 to November 26, 2019), and the daily Hash rates of Ethereum (from August 7, 2015 to November 26, 2019). The cryptocurrency price series is from the Coin Market Capitalization platform, the Bitcoin Hash rate series is from the Quandl platform,^3^ the Ethereum Hash rate is from the Etherscan platform,^4^ and the annual electricity demand series (from 2013 to 2019) is from the World Bank Indicator (WDI).

Following Goodkind et al. [9] electricity consumption is used to proxy or capture the electricity demand by the cryptocurrency markets. For the electricity demand series, the Denton-Cholette averaging conversion techniques of temporal disaggregation of a time series [[57], [58], [59], [60]] were used to convert it to a higher frequency (daily). This article employed the VAR(p) model, the nonparametric & parametric granger causality model, and the asymmetric BEKK volatility model. The parametric models are called the VAR−MGARCH−GJR−BEKK model.

### 3.2 Models

A key major limitation is the isolation of the actual electricity use for cryptocurrency mining and trading. Li et al. [10] used the thermal design power of CPUs and GPUs for the mining machines to capture the electricity use of cryptocurrency mining. However, this is just the design energy use and does not capture the actual electricity use. Another challenge is that these designs of thermal power are only based on the information shared online which may differ from the actual number of such machines used for their mining activities. Besides, the thermal design power does not capture the actual use of electricity for trading these cryptocurrencies used by other gadgets like phones, notepads, etc. More so, rapid increases in electricity use by most economies have been reported to be a result of cryptocurrency mining activities, examples are seen in China, Japan, Bangladesh, etc. As a result, most governments make tremendous efforts to cease and confiscate these mining machines. Therefore, the daily electricity consumption is used. This, however, does not completely isolate other factors affecting electricity use in these economies but within the increasing hash rate periods for these cryptocurrencies, the rapid electricity use changes are taken to be largely caused by the mining and trading of the cryptocurrencies. Similarly, to estimate the carbon emission impact of cryptocurrency mining on an economy, Goodkind et al. [9] also used the aggregate electricity usage and electricity generation emission rates (for the US and China) from sources like fossil fuel, nitrogen oxide, sulfur dioxide, etc to capture the carbon emission released to generate one cryptocurrency coin.

(1) $$A_{t}=\mu_{t}+a_{t}$$

$$\mu_{t}=E\left(A_{t}|F_{t-1}\right)\;and\;a_{t}|F_{t-1}∼MN\left(0,\Sigma \right)$$

(2) $$\phi_{i}\left(L,p_{i}\right)A_{it}=b_{i0}+b_{i1}t+a_{it}$$

To achieve the return spillover effects and granger causality objectives, the conditional mean equation for each bivariate system of equation is modelled using the vector autoregressive model (VAR(p)) as stated in equations (1) and (2). $A_{t}$ = ${\left\{A_{i}\right\}}_{2\times 1}$ is a 2×1 vector of a cryptocurrency and electricity demand or consumption at time t with (conditional) expected values, $\mu_{2\times 1,t}$ and an error vector, $a_{2\times 1,t}$. The error term, $a_{t}$, conditioned on past information set, $F_{t-1}$, is Gaussian distributed with a **zero** mean and Σ variance-covariance matrix. The optimal lag, k, for the best order estimation of the VAR(p) is selected based on the information set of Akaike Information Criteria (AIC). The individual quantile i’s unique $VAR\left(p_{i}\right)$ conditional mean model with an autoregressive variable parameter function $\phi_{i}$ of lag $p_{i}$ together with the intercept and trend components are shown in equation (2) and capable of dictating empirical mean spillover and linear Granger causality between the cryptocurrency and electricity markets. The null hypothesis for the basic Granger causality [61] lead-lag tests can be captured as

$$H_{01}:{\left\{\phi_{2j}|A_{1t}\right\}}_{j=1}^{p}=0$$

$$H_{02}:{\left\{\phi_{1j}|A_{2t}\right\}}_{j=1}^{p}=0$$

$A_{1t}$ is the cryptocurrency market return while $A_{2t}$ is electricity use. Against the alternative that at least one of the estimates of the bivariate VAR model, $\phi_{ij}$, is statistically different from zero. Literarily, $H_{01}$ states that the cryptocurrency returns do not granger cause electricity demand. In other words, the returns made from participating in cryptocurrency markets, mining included, do not drive the electricity demand. Hence, there is no need to include past information on the cryptocurrency return while forecasting electricity demand for each quantile. Similarly, $H_{02}$ states that electricity demand does not granger cause the cryptocurrency market return. However, this lead-lag structure is built upon linearity in the functional relationship between the electricity market (demand side) and the cryptocurrency markets. However, this might not be the case always, therefore, a nonparametric approach that explores the data-driven form of relationship between the series is adopted as developed by Diks and Panchenko [62] which modified the test statistic of Hiemstra and Jones [63] and used by Shen et al. [64]. The null hypothesis of return does not cause trade volume is

$$H_{03}:A_{1,t+1}\left|\left(A_{1t},A_{2t}\right)\;∼\;A_{1,t+1}\right|A_{1t}$$

$$H_{04}:A_{2,t+1}\left|\left(A_{1t},A_{2t}\right)\;∼\;A_{2,t+1}\right|A_{2t}$$

For equation (3), the conditional variance, $H_{t}$ = ${\left\{h_{ij}\right\}}_{2\times 2}$, is a conditional heteroscedasticity variance-covariance matrix of $A_{t}$ (or $a_{t}$ equivalently) at time t and can be directly modelled in equation (4) using the BEKK model [65]. To capture asymmetric [66] volatility spillover effects, this paper employs the MGARCH−GJR−BEKK(C,D) volatility model in equation (5) to capture the volatility spillover between the electricity and the cryptocurrency markets. Okorie and Lin [14] and several other studies [67,68] have adopted the MGARCH−GJR−BEKK(C,D) modelling approach to study volatility spillover.

at=Ht12zt

(3) $$H_{t}=D_{t}R_{t}D_{t}$$

(4) $$H_{t}=C^{T}C+\sum_{i=1}^{C}{X_{i}}^{T}a_{t-1}a_{t-1}^{T}X_{i}+\sum_{i=1}^{D}{Y_{i}}^{T}H_{t-i}Y_{i}$$

(5) $$H_{t}=C^{T}C+\sum_{i=1}^{C}{X_{i}}^{T}a_{t-1}a_{t-1}^{T}X_{i}+\sum_{i=1}^{D}{Y_{i}}^{T}H_{t-i}Y_{i}+\sum_{i=1}^{C}{Z_{i}}^{T}\eta_{t-1}\eta_{t-1}^{T}Z_{i}$$

$$m_{t}={D_{t}}^{-1}a_{t}$$

$$m_{t}∼\;MN\left(0,{\;R}_{t}\right)$$

$$\eta_{ij,t}=\max\left[0,-m_{t}\right]$$

Dt=diag{h1t12,h2t12}2×2 is the conditional standard deviation matrix. $R_{t}={\left\{a_{ij}\right\}}_{2\times 2}$ is the conditional correlation matrix. $m_{t}={\left\{a_{ij}\right\}}_{2\times 1}$ is the standardized error. Following Okorie and Lin [14] and Okorie [67,68], **C** is a 2×2 upper triangular that ensures the positive definiteness of $H_{t}$. $X={\left\{x_{ij}\right\}}_{2\times 2}$ is a 2×2 matrix capturing the arch effect and the short-term volatility spillover between the electricity and cryptocurrency markets. $Y={\left\{y_{ij}\right\}}_{2\times 2}$ is a 2×2 matrix which captures the long-term persistent (garch effect) and volatility spillover between these two markets and $Z={\left\{z_{ij}\right\}}_{2\times 2}$ is a 2×2 matrix that captures the markets' asymmetric information (positive and negative) effect with different influences on the markets. The asymmetric component is denoted as $\eta_{t}={\left\{\eta_{ij}\right\}}_{2\times 1}$. In null hypothesis forms, $x_{21}=y_{21}=0$ depicts that there is no volatility spillover from the electricity market (market 2) to the cryptocurrency market (market 1) while $x_{12}=y_{12}=0$ suggests no spillover of volatility from the cryptocurrency market (market 1) to the electricity market (market 2). While the null of no volatility spillover between the markets is stated as $x_{12}=y_{12}=x_{21}=y_{21}=0$ and $z_{12}=z_{21}=0$ is the null hypothesis for no asymmetric effect between the markets. These hypotheses are tested using the Wald statistic which is $X^{2}$ distributed. It is important to state that there are two broad approaches adopted in literature to study information spillover between and among markets. The first approach is that which is adopted in this paper which is capable of dictation substantial return and volatility transmission between two or among several markets. However, the leading information receiver or transmitter is not of interest. If that were to be our interest, the second approach, network system analysis would be adopted since it captures the overall information net transmitter or net receiver, and the leading net transmitter and receiver in the system [2].^5^

## 4 Results and discussion

Table 1 shows the basic summary statistics information of the return of Bitcoin, Ethereum, Bitcoin Hash rates (BTHashR), and Ethereum Hash rates (ETHashR) as well as the pre-estimation diagnostic test results of series. 2404 (1573) daily price values of Bitcoin (Ethereum) are used to compute the returns using the log difference approach. The return series both have approximately zero mean, negative skewness and kurtosis higher than the normal distribution. Thus, the return series are not normally distributed and have experienced extreme negative returns as supported by Pessa et al. [69]. On the other hand, the hash rates are positively skewed with kurtosis values less than that of a normal distribution. The Jacque Bera normality test result shows that the return series are not normally distributed. Intuitively, the return series should not be treated as white noise, using its unconditional mean and examining the conditional variance behaviour. However, they are to be fitted, to estimate their conditional mean before using their residuals to conduct the conditional variance analysis. The Ljung-Box linear and autocorrelation tests confirm that there is some information to extract from these return series using the conditional mean and conditional variance models. The return series is stationary at level forms while the Hash rates are stationary at the first difference with test statistics of −17.231 and −8.5242 for BTHashR and ETHashR respectively (see Table 2).

**Fig. 3:**
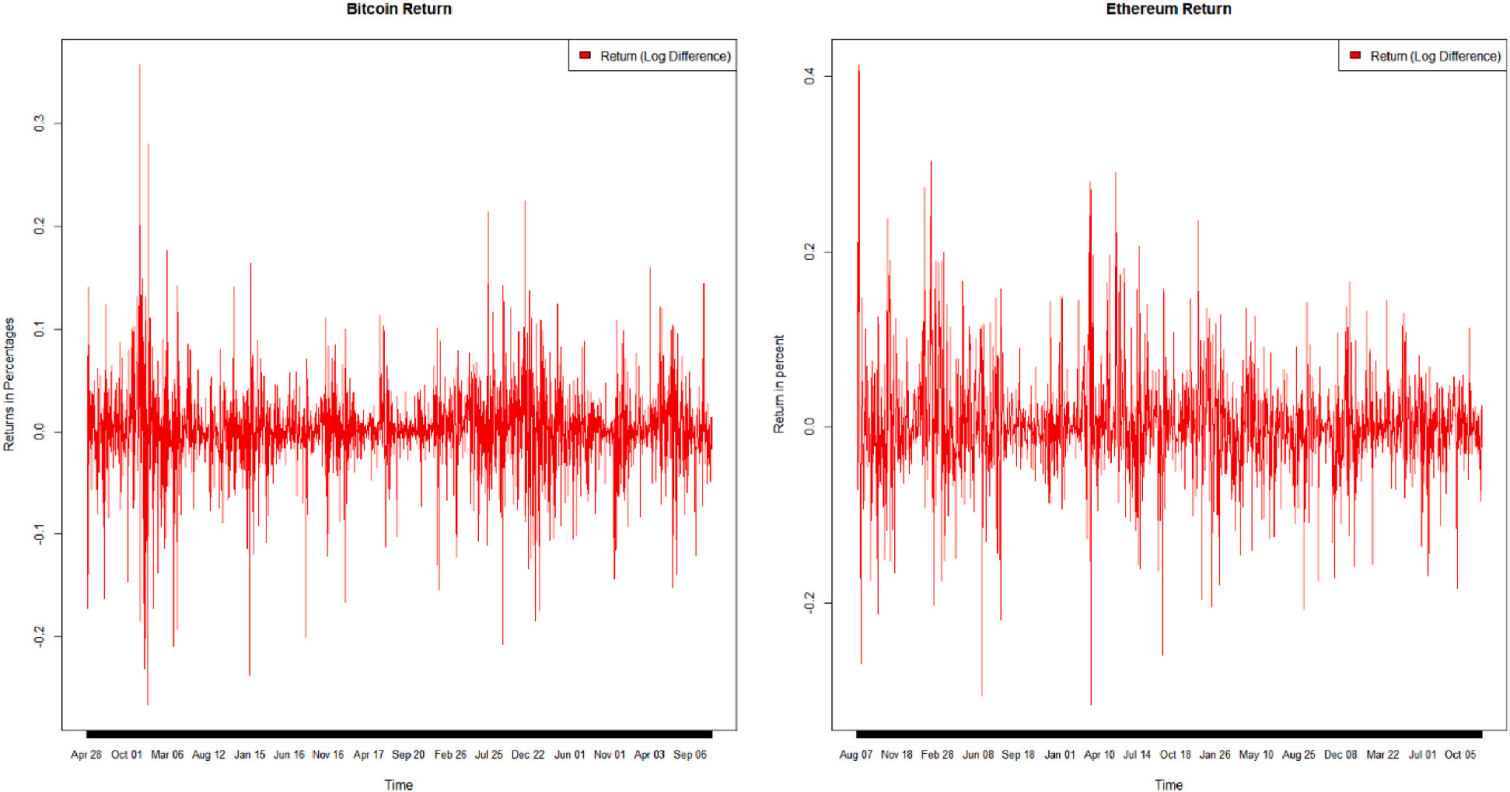
Returns - measured as closing price growth rates.

Figure 3 shows the trends of Bitcoin and Ethereum returns over time, within the sampled periods for both cryptocurrencies. The charts confirm that there have been reasonable amounts of positive and negative returns from investing in both markets over time. The sampled countries are grouped based on the electricity cost of mining a Bitcoin according to the findings of Reiff (2018). Let's say $C_{i}$ is the electricity cost for country i, Q25 is defined as the countries that satisfy $\$1,190\leq C_{i}\leq \$4,329$. Similarly, Q50, Q75, and Q100 satisfy $\$4,329<C_{i}\leq \$6,543$, $\$6,543<C_{i}\leq \$9,120$, and $\$9,120\leq C_{i}\leq \$26,170$ respectively.

Based on the electricity demand for economies in each category, an index is formed to represent the electricity demand for the group using the Principal Component Analysis (PCA). The first principal components for the four quantiles are used to represent the electricity demand for each group of countries based on the electricity cost of mining one Bitcoin, based on the findings of Reiff (2018). The amount of information (variations) captured by these first principal components are 50 %, 53.5 %, 79.5 %, and 61.2 % of the total variations of electricity demand for each stratum, respectively. It is very important to state that the original covariates employed in the computational analysis of these indexes are at their stationarity levels. This is a pre-condition for constructing indexes using time series i.e. the original p−dimensional covariates are stationary [70].

Table 2 shows the unit-root test results on the quantile electricity indexes, cryptocurrency prices, and Hash rates. Considering the Bitcoin sample length, the electricity indexes, Bitcoin price, and Hash rates are not stationary at the level form but at first difference. This is also the case for Ethereum prices and Hash rates but not for the electricity indexes. This is also the case while considering Ethereum sample size periods. On this note, the variables at the point of stationarity are used to carry out the proposed analysis. Table 3 presents the parametric and nonparametric Granger causality test of a lead-lag relationship between Bitcoin and Ethereum prices and their Hash rates to be able to ascertain whether mining activities drive or granger causes the price variations for the Bitcoin and Ethereum cryptocurrencies.

To avoid the probability of making higher type I errors, both the hash rates and the cryptocurrency prices were made stationary (i.e., differenced once) before conducting the lead-lag tests given they are stationary at first difference. From the results presented in Table 3, there is evidence of a lead-lag relationship from cryptocurrency prices (Bitcoin and Ethereum) to the Hash rates and from the Hash rates to the cryptocurrency prices and vice versa. Moreover, it is important to point out that the granger causality relationship from the bitcoin price to its Hash rate is not parametrically (linearly) but nonlinearly (nonparametrically) captured. Suggesting that these two series are nonlinearly related. As such, the bitcoin price can nonlinearly forecast its future hash rate. This result is intuitive and suggests the increasing activities of cryptocurrency miners, Bitcoin and Ethereum, significantly drive their prices and in return, these increasing prices also drive miners to mine more and even introduce new miners into the industry due to the incentives provided by the increasing prices.

The result in Table 3 is intuitively based on the fact that the quantity supplied is a key price determination factor (basic price mechanism knowledge). The mining of cryptocurrencies (shown by the hash rates) introduces new cryptocurrencies in the blockchain, thereby, instituting the supply side of the cryptocurrency markets. The bidirectional granger causality backs the fact that the increased cryptocurrency prices serve as an incentive for the cryptocurrency mining sector to develop and welcome new miners. Also, the increasing supply of cryptocurrency drives the price of cryptocurrencies and vice versa. For instance, given that crypto prices have been increasing in recent years; an increase in crypto prices as a result of increased supply (since mining granger causes crypto prices), it has to be the case that the demand outweighs the supply (excess demand) and when crypto prices decrease, the supply outweighs the demand (excess supply) and vice versa. This is very much the case as new cryptocurrency investors are joining the market now and then, conversely, old investors leave the markets too for losses and other personal reasons.

Table 4 presents the Granger causality test results between cryptocurrency returns (Bitcoin and Ethereum) and the demand for electricity at the different levels of quantiles of electricity rate or costs. Based on the results presented in Table 5 and on the linear bivariate VAR parametric Granger causality test results (the variables are at stationarity levels), there exists a significant Granger causality from Bitcoin returns to the Electricity Demand on a global perspective. This is also the case for the bottom 25 % quantile, 75 % quantile, and top 100 % quantile of electricity cost groupings. This is intuitive in the sense that the increasing prices and return of Bitcoin have led to increased mining of Bitcoins and thus, increased demand for electricity in these countries. However, there is no statistical evidence of any directional causality from Ethereum returns on the electricity demand. This is, however, the case since Ethereum is still not a well-developed and traded cryptocurrency and its price is still far below that of Bitcoin and other cryptocurrencies and as such might not be capable of generating enough return for active investors and miners. Next, this article constructed an aggregate or say global electricity index for the sampled 89 countries, as a robustness check, to examine the lead-lag relationship between cryptocurrency returns (Bitcoin and Ethereum) and the electricity demand. The results in Table 4 are intuitive and show strong evidence that the cryptocurrency (Bitcoin) market return drives the demand or consumption of electricity in countries with the least electricity cost, given that miners tend to relocate or establish their mining outlets in such economies and countries with high electricity costs.

It is direct and understandable that given high returns from the Bitcoin market, Bitcoin miners tend to move to regions with lesser electricity costs (assuming free movement without any or with reasonable and affordable number of restrictions) to set up mining outlets. However, our results also show that Bitcoin's return drives the demand for electricity even in the regions with higher electricity costs for mining a Bitcoin. These economies include the likes of China, the USA, Japan, etc. The return from the Bitcoin market still drives the demand for electricity in these economies because the cryptocurrency (Bitcoin) market is well developed in these economies relative to other economies (even the lower mining electricity cost countries). This is entirely true given that the early exchange markets of cryptocurrencies started in these economies (such as Japan, the United States of America, China, etc.) and the history of cryptocurrency mining stems from these economies too. These facts are the reasons behind miners situating their mining outlets in these economies despite the higher electricity costs of mining cryptocurrencies. The results are presented in Table 5. These results also confirm the existence of an uni-directional Granger causality running from the Bitcoin return to the Demand for Electricity on a global level. In other words, there is empirical evidence of the returns from the Bitcoin markets being the reason behind the increased demand for electricity which is used for active trading on the exchanges as well as mining Bitcoins. Converse to the lead-lag relationship results between the cryptocurrencies and their hash rates in Table 3, the results in Table 4 and Table 5 show that the lead-lag relationships between cryptocurrencies return (particularly the bitcoin) and the demand for electricity is parametric and linear against a nonlinear or nonparametric relationship. Therefore, the bitcoin returns can linearly forecast the electricity demand on a global level and for the different country groupings or strata.

Furthermore, the paper examines volatility spillover between the cryptocurrency markets (Bitcoin and Ethereum) and the electricity markets (Demand side) using the conditional model, VAR−MGARCH−GJR−BEKK(p,1,1). The optimal lag length order for all conditional mean models in these analyses, p, is based on the selection of the Akaike Information Criteria (AIC). Conversely, the conditional heteroscedasticity variance equation models are fixed to order one for the short-term, long-term, and asymmetric components of the model. Upon estimating this model for each group of countries, the Wald test is conducted and the result is presented in Table 6 based on the BEKK(1,1) estimation results. Based on this result, there exists a significant level of volatility spillover from the demand for electricity to the cryptocurrency markets (Bitcoin and Ethereum) at all quantiles or strat or country groupings. In other words, irrespective of the electricity rate an economy charges, rapid fluctuations in the demand for electricity or electricity demand volatility transmits to the cryptocurrency markets, as evidenced by the Bitcoin and Ethereum markets. This is also intuitive given that researchers and energy specialists have warned and tried to draw the attention of the world towards the increased demand for electricity for crypto mining as this is the present situation even when economies want to reduce electricity consumption and carbon emissions and/or divert to cleaner energy sources. On this note, this paper presents evidence of this claim given the lead-lag relationships and volatility spillover between the cryptocurrency markets (Bitcoin and Ethereum) and the Demand for Electricity.

The results in Table 6 show that while there is significant volatility spillover from the electricity market (demand side) to the cryptocurrency markets, there is no evidence of significant volatility from the cryptocurrency markets to the electricity market (demand side). Intuitively, volatility measures how much the price or return (demand) changes for the cryptocurrency (electricity) market. Therefore, the rate of electricity demand changes in the electricity market transmits to the rate of cryptocurrency return changes and not vice versa. In other words, this result shows while the rate of electricity consumption changes transmits to the cryptocurrency markets, the rate of cryptocurrency return changes does not spill over to the electricity market. This implies that (the demand for electricity for) crypto mining activities does not significantly transmit into the electricity market. This is due to the development of different versions of cryptocurrency mining machines for electricity consumption efficiency. The sole aim of the inversion of new mining machines is to reduce the consumption of electricity for mining activities. Sutherland [71] noted that the recent versions of mining machines significantly reduce energy consumption and improve efficiency. For instance, the GTX 980 Ti consumes a whole lot of energy to mine one coin relative to the GTX1070^6^ and GTX 1080. Similarly, Bitmain's Antminer S7 consumes a minimum of 1293 W at room temperature (and positively correlated with the room temperature). Bitmain's Antminer latest version, T17+, is more electricity consumption efficient relative to T17e, T17, S5, R4, S17e, Canaan's AvalonMiner 1146, etc. Nowadays, Bitcoin mining is done using ASICs (Application Specific Integrated Circuits) machines that have lower electricity costs (higher electricity consumption efficiency). More so, the number of mining hardware, rate of utilization, efficiency level, etc. contribute to the reason there is no significant evidence of volatility spillover from the crypto market to the electricity market. In further comparison with existing studies, the findings that the cryptocurrency returns significantly forecast the electricity market's demand presents more evidence in support of the forecastability of the electricity market prices [13]. This, in turn, gives flesh to the cryptocurrency mining and carbon emission claims of Liu et al. [11]. In general, the findings of this study confirm the existence of significant nexus between the cryptocurrency market and the electricity market [12], even on a global level.

## 5 Policy implications and conclusion

### 5.1 Policy implications

Generally, the policy implications of this study stem directly from the empirical results and modelling strategies used to elicit these findings (or substantial patterns) from the observed data information set. To recap the major objectives investigated in this study; firstly, this study investigates both linear (parametric) and non-linear (non-parametric) directional (bidirectional and unidirectional) Granger causality between the hash rates and the cryptocurrency (Bitcoin and Ethereum) prices. Secondly, the parametric and non-parametric directional lead-lag relationship between electricity demand and the cryptocurrency returns in eighty-nine (89) active cryptocurrency trading and mining economies are examined. Furthermore, this lead-lag relationship existing between the demand for electricity and the cryptocurrency returns is disaggregated into sub-groups (strata) of economies based on their electricity cost. Finally, we examined the spatial information spillovers between the electricity market and the cryptocurrency market (Bitcoin and Ethereum). Based on our findings the following policy implications are deduced and drawn to benefit the end-users of this study such as the governments, policymakers at different levels of the government, energy (electricity) market authorities, institutional and private cryptocurrency investors, cryptocurrency active miners and traders, etc.

1. Given the substantial bidirectional lead-lag relationship existing between the cryptocurrency prices and hash rates, both for Bitcoin and Ethereum, policymakers and investors could predict, as well as forecast, the patterns in the cryptocurrency (Bitcoin and Ethereum) prices (hash rates) using the observed past information set of the hash rates (cryptocurrency prices). It is important to state that this lead-lag relationship exists both when a parametric linear functional form is assumed between these variables and when non-linear non-parametric functional relationships are assumed to exist between them. To drive home this implication to the end-users, let's take investors for instance, both private and institutional investors aim to beat the market. In other words, they aim to be able to foretell or foresee future market movements. This is always the goal for every investor as this would help the investors to take the right action in the current period (by either taking long or short positions) to make profits from the foretold market movements. To achieve this target, our study shows that past and current period hash rates (price) of Bitcoin and Ethereum can significantly foretell the future movement or pattern of their prices (hash rates). This implies that investors could monitor and use the observed hash rates of Bitcoin and Ethereum to foretell the future directional movement of Bitcoin and Ethereum prices for taking either short or long positions to make profits from this foretold pattern. This is one of the practical implications of this finding to both institutional and private investors as one of the end-users of this study.
2. Moreso, the revelation that Bitcoin's return significantly predicts the electricity demand for most of the strata (country groups) is very intuitive and has substantial practical implications. This finding is confirmed both for the economies with the least and highest electricity rate cost. This implies that as the cryptocurrency (Bitcoin) market develops and yields more returns, the electricity demand, from the economies where Bitcoin is actively traded and mined, substantially increases. Therefore, this increase in the returns from the Bitcoin market is a signal for the preceding increase in the electricity demand, hence, policymakers are presented with the opportunity to take the right actions to achieve their set targets. For instance, let's assume an economy wishes to generate more internal revenue, perhaps from the electricity market, the policymakers can directly increase the electricity rate given the increases in the return (price) of Bitcoin. This will create a new equilibrium point in the domestic electricity market, higher than the old equilibrium point and most likely would generate more revenue for the economy. Alternatively, if the economy is targetting a substantial decrease in electricity demand, the signalling attribute of the Bitcoin price or return increases suggests that the authorities could introduce special mining and trading charges, aimed at reducing the active mining (and trading) activities of Bitcoin. Conversely, the authorities could fund and embark on cleaner energy (electricity) sources that these miners could subscribe to for cleaner sources of electricity while they actively mine and trade these coins and vice versa.
3. The immediate discussed implication is on disaggregated strata levels; for similar electricity rate economies. However, a global perspective of the lead-lag relationship between the returns from the cryptocurrency markets (Bitcoin and Ethereum) and the electricity markets is also investigated. On a global basis, there is also, significant evidence of a unidirectional lead-lag relationship from the Bitcoin market to the global electricity demand. This implies that given boom periods in the Bitcoin markets, economies of the world, on average, experience an increase in electricity demand. This is very intuitive given that miners can easily move from economies with high electricity rates to lower electricity rate economies. In other words, no economy or government is saved from the Bitcoin market boom via its electricity demand. Therefore, every economy should also play its role in curtailing the electricity demand when it implies more emissions leading to global warming, and support cleaner sources of electricity such that the increased demand for electricity by the miners would not affect humanity adversely. Furthermore, incentives could be provided to enable the shift of electrical energy supplies, to miners, to cleaner energy (electricity) sources and technologies.
4. Last but not least, there exists substantial volatility information spillover between the cryptocurrency markets and the electricity markets. This implies that the electricity (cryptocurrency) markets are not isolated or immune from shocks or events that are capable of causing rapid swings or fluctuations in the cryptocurrency (electricity) market as these swings or volatilities in any of these markets can transmit or spill over to the other market. This further confirms the connectivity between the electricity or energy markets and the cryptocurrency markets. Therefore, policymakers at all levels of the government and electrical energy authorities could monitor the swings and rate of price changes in the cryptocurrency markets, and take proactive measures to counteract the anticipated impact or swings in the electricity market through the development and application of the right policies and vice versa.

Some other policy implications, for the end-users of this study, can be deduced from our methods and findings. However, we only present most of the key implications of this study to the end-users.

### 5.2 Conclusion

This paper sets out to examine the lead-lag relationships that exist between cryptocurrency prices and their Hash rates using the Bitcoin and Ethereum markets. Besides, this paper also studies the lead-lag and volatility spillover relationships between the demand for electricity and cryptocurrency returns using the electricity demand of 89 countries and the returns of Bitcoin and Ethereum respectively. The results confirm a bidirectional relationship between the cryptocurrency prices and the Hash rates as well as a unidirectional Granger causality from Bitcoin return to electricity demand and a significant volatility spillover from the electricity market (demand side) to the Bitcoin market. Based on these findings, the concerned bodies should look into the increasing demand for electricity for cryptocurrency trading and mining as well as the need to reduce carbon emissions (global warming) and use cleaner energy sources. A holistic view would inform these bodies of possible policies to put in place that would mitigate any future contingencies. The mining of cryptocurrencies inevitably requires a huge amount of electricity demand. For expansion and prospects in fresh regions such as India, Nigeria, Ghana, etc. There is a pressing need to explore cleaner energy sources with reduced or no carbon emission as the source of power for mining cryptocurrencies since the emission of carbon from electricity consumption is the major concern of every economy towards mitigating global warming and environmental degradation. Besides, the lockdown and stay-at-home effects of the COVID-19 pandemic in most economies invariably could lead to more electricity demand for mining cryptocurrencies from the comfort of one's home amongst other uses of electricity during this pandemic; as long as he/she has the mining equipment.

## Ethics declaration

• Review and/or approval by an ethics committee was not needed for this study because no laboratory experiments were conducted with the students (respondents) and no human experimental data was used for analysis in this study.
• Informed consent was not required for this study because no laboratory experiments were conducted with the students (respondents) and no human experimental data was used for analysis in this study.

## Data availability statement

1. **Data Availability:** Sharing research data helps other researchers evaluate your findings, build on your work and to increase trust in your article. We encourage all our authors to make as much of their data publicly available as reasonably possible. Please note that your response to the following questions regarding the public data availability and the reasons for potentially not making data available will be available alongside your article upon publication. Has data associated with your study been deposited into a publicly available repository? – NO.
2. Please select why. Please note that this statement will be available alongside your article upon publication. as follow-up to "**Data Availability.** Sharing research data helps other researchers evaluate your findings, build on your work and to increase trust in your article. We encourage all our authors to make as much of their data publicly available as reasonably possible. Please note that your response to the following questions regarding the public data availability and the reasons for potentially not making data available will be available alongside your article upon publication. Has data associated with your study been deposited into a publicly available repository? – Data will be made available on request

## CRediT authorship contribution statement

**David Iheke Okorie:** Writing – review & editing, Writing – original draft, Software, Methodology, Investigation, Formal analysis, Data curation, Conceptualization. **Joel Miworse Gnatchiglo:** Writing – original draft, Resources, Methodology, Investigation, Data curation, Conceptualization. **Presley K. Wesseh:** Writing – original draft, Resources, Investigation, Formal analysis, Data curation, Conceptualization.

## Declaration of competing interest

The authors declare that they have no known competing financial interests or personal relationships that could have appeared to influence the work reported in this paper.

## Figures and Tables

**Table 1:** Descriptive Pre-estimation Statistics for Bitcoin and Ethereum (returns and hash rates)

Statistic	Bitcoin	Ethereum	BTHashR	ETHashR
Obs.	2403	1572	2404	1573
Mean	0.0017	0.0025	15469628	108820.5
Variance	0.0018	0.0052	5.971e+14	1.029e+10
Skewness	−0.1582	−3.4137	1.6858	0.3137
Kurtosis	7.6632	70.5500	2.0528	1.3841
Jacque Bera	5889.9***	3291***	1560.8***	151.36***
LB-Q (10)	25.792***	23.004***	23074***	15665***
LB-Q^2^ (10)	504.13***	33.547***	21635***	15635***
ADF @ I(0)	−11.425***	−10.709***	1.2063	−0.71905

Notes: Jarque-Bera statistic that tests for the null hypothesis of a normal distribution. LB-Q(10) and LB-Q^2^(10) are Ljung-Box test statistics of the returns series for first and higher-order no autocorrelation tests, up to lag order of 10 serial autocorrelations, respectively. Ljung Box is a portmanteau univariate statistic test that could also serve as a test of conditional heteroscedasticity in the null hypothesis following Tsay (2014).

∗∗∗ p-value <0.01; **p-value <0.05; *p-value <0.1.

**Table 2:** Stationarity tests for Electricity Index, Crypto Prices & Hash Rates

Indexes	I (0)	I (1)	I (0)	I (1)
	**w/Bitcoin** (April 28, 2013 - November 26, 2019)		**w/Ethereum** (August 7, 2015 - November 26, 2019)	
Q25	−3.9359**		−3.9037**	
Q50	−3.4282**		−4.5513***	
Q75	−3.6563**		−4.6815***	
Q100	−3.8479**		−4.2016***	
Prices	−2.7712	−12.935***	−1.7351	−12.714***
Hash Rate	1.2063	−17.231***	−0.7191	−8.5242***

∗∗∗ p-value <0.01.

∗∗ p-value <0.05; *p-value <0.1.

**Table 3:** Lead-lag Relationship Tests between Crypto Prices & Hash Rates

Method	Null Hypotheses	Statistic	P-Value	Conclusion
Parametric	Bitcoin Hash Rate does not granger cause its Price	4.303***	0.0000	Reject
Nonparametric		8.776***	0.0000	Reject
Parametric	Bitcoin Price does not granger cause its Hash Rate	0.721	0.6543	Fail to Reject
Nonparametric		6.899***	0.0000	Reject
Parametric	Ethereum Hash Rate does not granger cause its Price	2.592**	0.0167	Reject
Nonparametric		7.206***	0.0000	Reject
Parametric	Ethereum Price does not granger cause its Hash Rate	4.765***	0.0000	Reject
Nonparametric		6.055***	0.0000	Reject

∗∗∗ p-value <0.01.

∗∗ p-value <0.05; *p-value <0.1. Bandwidth = 0.5 and embedding dimension = 2.

**Table 4:** Heterogeneous lead-lag relationship between Crypto Return & Electricity Demand

Method	Null Hypotheses	Statistic	P-Value	Conclusion
	Q25 countries			
Parametric	Bitcoin return does not granger cause Elec. Demand	5.453***	0.0000	Reject
Nonparametric		0.498	0.6908	Fail to Reject
Parametric	Ethereum return does not granger cause Elec. Demand	0.262	0.8531	Fail to Reject
Nonparametric		0.285	0.3880	Fail to Reject
	**Q50 countries**			
Parametric	Bitcoin return does not granger cause Elec. Demand	0.161	0.8513	Fail to Reject
Nonparametric		0.396	0.6540	Fail to Reject
Parametric	Ethereum return does not granger cause Elec. Demand	0.336	0.7149	Fail to Reject
Nonparametric		0.435	0.6681	Fail to Reject
	**Q75 countries**			
Parametric	Bitcoin return does not granger cause Elec. Demand	4.560***	0.0000	Reject
Nonparametric		0.349	0.3636	Fail to Reject
Parametric	Ethereum return does not granger cause Elec. Demand	0.2702	0.7633	Fail to Reject
Nonparametric		0.122	0.4515	Fail to Reject
	**Q100 countries**			
Parametric	Bitcoin return does not granger cause Elec. Demand	4.666***	0.0000	Reject
Nonparametric		1.173	0.2224	Fail to Reject
Parametric	Ethereum return does not granger cause Elec. Demand	0.429	0.6514	Fail to Reject
Nonparametric		0.234	0.4134	Fail to Reject

∗∗∗ p-value <0.01; **p-value <0.05; *p-value <0.1. Bandwidth = 0.5 and embedding dimension = 2.

**Table 5:** Lead-lag relationship between Electricity & Cryptocurrency on a global basis

Method	Null Hypotheses	Statistic	P-Value	Conclusion
Parametric	Bitcoin return does not granger cause Elec. Demand	3.341***	0.001	Reject
Nonparametric		0.971	0.1658	Fail to Reject
Parametric	Ethereum return does not granger cause Elec. Demand	0.325	0.7227	Fail to Reject
Nonparametric		0.236	0.3427	Fail to Reject

∗∗∗ p-value <0.01; **p-value <0.05; *p-value <0.1. Bandwidth = 0.5 and embedding dimension = 2.

**Table 6:** Wald Statistic's volatility Spillover effects between electricity & cryptocurrencies

	Null Hypotheses	Statistic	P-Value	Conclusion
Q25 countries	Elec. Dd. has no volatility spillover on Bitcoin Mkt	152.6***	0.0000	Reject
	Bitcoin Mkt. has no volatility spillover on Elec. Dd.	0.002	0.6506	Fail to Reject
	Elec. Dd. has no volatility spillover on Ethereum Mkt	279.6***	0.0000	Reject
	Ethereum Mkt. has no volatility spillover on Elec. Dd.	0.003	0.9559	Fail to Reject
Q50 countries	Elec. Dd. has no volatility spillover on Bitcoin Mkt	187.5***	0.0000	Reject
	Bitcoin Mkt. has no volatility spillover on Elec. Dd.	0.001	0.9908	Fail to Reject
	Elec. Dd. has no volatility spillover on Ethereum Mkt	157.2***	0.0000	Reject
	Ethereum Mkt. has no volatility spillover on Elec. Dd.	0.0003	0.9853	Fail to Reject
Q75 countries	Elec. Dd. has no volatility spillover on Bitcoin Mkt	230.4***	0.0000	Reject
	Bitcoin Mkt. has no volatility spillover on Elec. Dd.	0.008	0.9836	Fail to Reject
	Elec. Dd. has no volatility spillover on Ethereum Mkt	625.3***	0.0000	Reject
	Ethereum Mkt. has no volatility spillover on Elec. Dd.	0.001	0.9709	Fail to Reject
Q100 countries	Elec. Dd. has no volatility spillover on Bitcoin Mkt	592.1***	0.0000	Reject
	Bitcoin Mkt. has no volatility spillover on Elec. Dd.	0.035	0.8507	Fail to Reject
	Elec. Dd. has no volatility spillover on Ethereum Mkt	***	0.0000	Reject
	Ethereum Mkt. has no volatility spillover on Elec. Dd.	0.0009	0.9809	Fail to Reject

∗∗∗ p-value <0.01; **p-value <0.05; *p-value <0.1.

## Footnotes

1 See https://www.statista.com/statistics/280704/world-power-consumption/.

2 The 89 countries are grouped into different homogenous groups or strata based on their electricity rates (Reiff, 2018). Secondly, we construct electricity consumption indexes for these homogenous groups which are directly used in the analysis. The countries are Albania, Canada, India, Kuwait, Saudi Arabia, United Arab Emirates, Bangladesh, China, Iran, Myanmar, Serbia, Uzbekistan, Belarus, Egypt, Kazakhstan, Nepal, Suriname, Zambia, Bosnia & Herzegovina, Georgia, Kosovo, Paraguay, Ukraine, Argentina, Gibraltar, Malaysia, Russia, United States, Brunei Darussalam, Hungary, Moldova, Slovak, Vietnam, Bulgaria, Iceland, Nigeria, Sweden, Croatia, Indonesia, Peru, Thailand, Estonia, Lithuania, Romania, Turkey, Brazil, Hong Kong, Latvia, Montenegro, Philippines, South Africa, Colombia, Iraq, Luxemburg, New Zealand, Poland, Switzerland, Finland, Israel, Malta, Norway, Singapore, France, Jamaica, Mexico, Pakistan, Slovenia, Australia, Curacao, Ireland, Nicaragua, United Kingdom, Bahrain, Cyprus, Italy, Portugal, Uruguay, Belgium, Denmark, Japan, Spain, Cambodia, Germany, Jordan, Sri Lanka, Chile, Greece, Netherlands, and Trinidad & Tobago.

3 See https://www.quandl.com/data/BCHAIN/HRATE-Bitcoin-Hash-Rate.

4 See https://etherscan.io/chart/hashrate.

5 For univariate cases, mit=|ait|hit∼N(0,1)

6 GTX 1070 remains the most efficient in terms of energy consumption. See crypto mining blog (http://cryptomining-blog.com/tag/power-consumption/).
